# Calcific tendonitis of the shoulder; when should we operate?

**DOI:** 10.1308/rcsann.2025.0046

**Published:** 2025-07-15

**Authors:** T Berg, JM Bentley, N Saravanan, H Morris, AP Dekker, DI Clark

**Affiliations:** ^1^ University of Nottingham, UK; ^2^ Royal Derby Hospital, UK; ^3^ Pulvertaft Hand Centre, Royal Derby Hospital, UK; ^4^ University Hospitals of Derby and Burton NHS Foundation Trust, UK

**Keywords:** Calcific tendonitis, Calcification, Shoulder, Arthroscopy, Surgery, Nonoperative management

## Abstract

**Introduction:**

This study aimed to determine patient factors associated with the failure of nonoperative management of calcific tendonitis and subsequent operative intervention. The secondary aim was to assess whether the size of the calcific deposit can determine the need for surgery, as shown by previous studies in the literature.

**Methods:**

A retrospective review of a prospectively maintained database of a consecutive series of patients diagnosed with calcific tendonitis attending a single hospital trust orthopaedic department between 2014 and 2018 was performed. Data were collected on the size and location of the calcium deposit, comorbidities, Oxford Shoulder Score and functional range of movement.

**Results:**

A total of 61 patients were included. Factors associated with the failure of nonoperative management were: size of calcific deposit >10mm (*p*=0.009), female sex (*p*=0.005), a chronic condition of more than eight months duration (*p*=0.001), failed previous treatment (still symptomatic after previous management, requiring treatment) (*p*=0.001) and patients for whom steroid injections did not control their symptoms (*p*=0.02).

**Conclusions:**

Patients with a calcific deposit larger than 10mm, who are female, or have had symptoms for more than eight months are more likely to require surgery. Those with a transient response to steroid injections and or physiotherapy are also more likely to require surgical management.

## Introduction

Calcific tendonitis of the shoulder is a painful and debilitating condition characterised by calcific deposits that form within the rotator cuff tendons.^1^ It has a prevalence of 2.7–20%,^2–4^ with approximately 50% of patients eventually becoming symptomatic.^5^ Calcific tendonitis can progress to cause several complications, including chronic pain, adhesive capsulitis, rotator cuff tears, greater tuberosity osteolysis and ossifying tendinitis.^6^

Risk factors for the development of calcific tendonitis have been shown previously to include increasing age, diabetes, smoking, sedentary occupation and female sex.^4,7,8^ Management may be nonoperative with analgesics or steroid injections and physiotherapy, with the literature describing variable success.^9^ Surgical treatment consists of arthroscopic debridement of the calcific lesions with or without subacromial decompression and rotator cuff repair.^10–12^

To our knowledge, there have been no published studies that demonstrate the clinical factors associated with the failure of nonoperative management, and the subsequent need for surgery in patients with calcific tendonitis. There are, however, two previous studies that investigate how radiological factors can influence the need for surgery. Ogon *et al* identified bilateral disease, disease localized anterior to the acromion, subacromial extension and high-volume calcific deposits as risk factors that made surgical intervention more likely.^13^ More recently, Drummond *et al* identified calcific lesions with a size greater than 10mm as a risk factor for operative intervention.^14^

This study aimed to determine patient factors associated with the failure of nonoperative management of calcific tendonitis and subsequent operative intervention. The secondary aim was to assess whether the size of the calcific deposit can determine the need for surgery, as shown by previous studies in the literature.^13,14^

## Methods

This study was registered through the hospital audit office as a service evaluation; therefore, ethical approval was not necessary.

**Figure 1:**
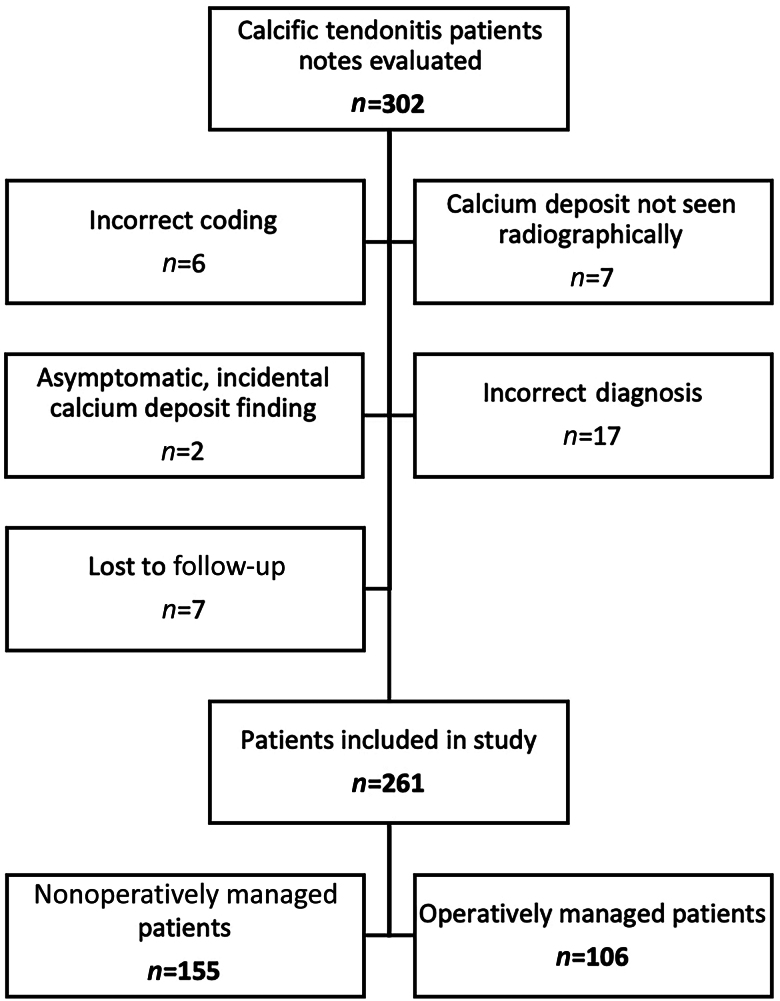
Flowchart displaying how patients with calcific tendonitis patients were included in study

A retrospective review of a prospectively maintained database was undertaken to identify a consecutive series of patients diagnosed with calcific tendonitis between 2014 and 2018 presenting to the Orthopaedic department at a single centre. Patients were diagnosed with calcific tendonitis if they were symptomatic, and had imaging (a mixture of x-ray, ultrasound and magnetic resonance imaging was used) that confirmed the diagnosis. The size of the largest deposit was recorded, and used as the ‘deposit size’ for that patient. The size of the deposit was measured by using the most accurate imaging modality available. Patients were excluded if calcific deposits were an incidental finding in asymptomatic patients, or if they were lost to follow-up (Figure 1).

The case notes and imaging of patients were reviewed, and an additional questionnaire was sent out by mail. This enabled extensive data to be collected, which included deposit size and location within the rotator cuff, patient age, sex, occupation type (sedentary, manual, not working or retired), ethnicity, body mass index (BMI), comorbidities, symptom onset, associated conditions, Oxford Shoulder Score (OSS) before and after treatment, previous management, range of movement (forward flexion (FF) and external rotation (ER)), length of any conservative management, length of follow-up, surgical intervention details and any complications. The questionnaire sent to patients is located in Appendix A.

Functional outcome was assessed using the OSS and range of movement (FF and ER) by the assessing clinician at follow-up appointments. The OSS is a validated scoring system used to score shoulder disability and pain, providing a score between 0 and 48.^15^

### Treatment algorithm

Patients who were reviewed in clinic with a diagnosis of calcific tendonitis had an initial OSS, and range of FF and ER scores taken (incoming scores). Patients were then referred to physiotherapy for an intensive course of rehabilitation and advice on the use of simple analgesia. Nonoperative adjuncts such as steroid injection and needling could also be used here.

After the course of physiotherapy and nonoperative management, patients were then reviewed back in the clinic, where patient symptoms were reviewed, and postconservative scores (OSS, ER and FF) were taken. At this point, a conversation would be had regarding whether their symptoms had improved and whether they were happy with conservative management, at which point they were discharged from the clinic, or whether symptom relief had not improved sufficiently and surgical intervention was required.

These patients who went on to have surgery were then reviewed in clinic following their operative intervention and postsurgery scores (OSS, ER and FF) were recorded.

By managing patients in this way, we were able to compare patients’ initial functional scores with the postconservative management scores for both groups (those who had responded to nonoperative management and those who had not, and therefore required further intervention). Subsequently, we were then able to compare postsurgery scores for patients who underwent this intervention.

### Surgical approach

All patients requiring surgery had an arthroscopic debridement – the gold standard treatment for calcific tendonitis.^16,17^ At the time of surgery, depending on the patient’s symptoms and imaging findings, additional procedures, such as rotator cuff repair, acromioclavicular joint (ACJ) excision, bursectomy, subacromial decompression and capsular release were performed. These additional surgeries were performed based on the patient’s symptoms and imaging findings. Intraoperative x-ray guidance was used in 46 of the surgeries – this depended on the need for it based on surgeon preference and complexity of surgery.

### Statistical analysis

Statistical analysis was performed using SPSS version 23.0 (IBM Corp, New York, NY, US). The Pearson chi-square test and Student’s *t*-test were used to analyse the data for categoric and continuous data, respectively. A *p*-value of <0.05 was considered statistically significant.

## Results

A total of 261 patients with calcific tendonitis met the inclusion criteria for the study. How these were identified is outlined in Figure 1.

### Patient demographics and comorbidities

Table 1 demonstrates the included patient demographics, associated comorbidities and symptomatic factors, including chronicity of symptoms. Significantly more women were treated surgically than men (*p*=0.005).

**Table 1:** Symptomatic factors with demographic and comorbid data

Patient demographic and comorbidities	Frequency			*p*-value	Statistical test
	Total analysed	Surgical	Conservative		
Mean age (SD)	52 (11)	51 (10)	53 (11)	0.098	Student’s *t*-test
Sex (male:female)	98:163	29:77	69:86	**0**.**005**	Pearson chi-square test
Occupation	173	76	97	0.594	Pearson chi-square test
Manual	84	35	49		
Sedentary	61	26	35		
Unemployed	15	7	8		
Retired	13	8	5		
Ethnicity	259	106	153	0.094	Pearson chi-square test
White British	206	90	116		
Asian	9	1	8		
White	7	3	4		
Black	6	3	3		
Other	7	1	6		
Mixed	2	2	0		
Not stated	22	6	16		
Mean BMI (SD)	30 (7)	31 (7)	29 (7)	0.155	Student’s *t*-test
Diabetic	239	16	20	0.69	Pearson chi-square test
Smoking (yes:no:ex-smoker)	41:108:37	22:49:11	19:59:26	0.096	Pearson chi-square test
Hypothyroidism (yes:no)	17:223	6:94	11:129	0.580	Pearson chi-square test
Hyperthyroidism (yes:no)	3:240	2:100	1:140	0.377	
Other thyroid and parathyroid pathologies (yes:no)	3:237	1:99	2:138	0.768	Pearson chi-square test
Hypercholesterolaemia/hyperlipidaemia (yes:no)	22:218	13:87	9:131	0.082	Pearson chi-square test
Vitamin D deficiency (yes:no)	11:229	4:96	7:133	0.715	Pearson chi-square test
Chronicity of symptoms	247	98	149	**<0**.**001**	Pearson chi-square test
Acute (<6 weeks)	62	11	51		
Subacute (6 weeks to 3 months)	15	5	10		
Chronic (3–7 months)	58	21	37		
More than 8 months	112	61	51		
Mean length of follow-up (SD)		30.75 (35.45)			
Mean pretreatment OSS (SD)	24.0 (8.7)	22.5 (8.0)	25.2 (9.1)	0.079	Student’s *t*-test

BMI = body mass index; OSS = Oxford shoulder score; SD = standard deviation

The need for surgery in patients with hypercholesterolaemia/hyperlipidaemia and BMI above 30, were not found to be statistically significant (*p*=0.082, *p*=0.066, respectively).

### Calcific deposit

The effect of the size of the calcium deposit on the need for surgical intervention is demonstrated in Table 2. Calcific deposit size was significantly larger in the surgical group (*p*=0.003) with a mean size of 12.462mm versus 10.170mm in the nonoperative group.

**Table 2:** Frequency of deposits greater than 1cm compared with less than 1cm in surgical and nonoperative groups (chi-square (*p*=0.009))

Deposit size	Surgical patients, n (%)	Nonoperative patients, n (%)	Total
<1cm	40 (15%)	83 (32%)	123
≥1cm	66 (25%)	70 (27%)	136
Total	106	153	259

Of the 106 of patients who were treated surgically, 67 had a deposit >10mm, whereas 71 of 155 of conservative patients had a deposit >10mm (*p*=0.005).

### Predictive symptoms for failure of nonoperative management

Table 3 outlines the nonoperative interventions that the patients included received. Those who underwent nonoperative management had more subacromial injections during their nonoperative treatment than those who went on to require surgical intervention (*p*=0.02). Patients receiving treatment in primary care (from a general practitioner and/or a physiotherapist) were more likely to require surgical intervention (*p*=0.001).

**Table 3:** Conservative treatment factors

Conservative treatment factors	Frequency			*p*-value	Statistical test
	Total analysed	Surgical	Conservative		
Previous treatment (yes:no)	112:135	62:37	50:98	<0.001	Pearson chi-square test
Subacromial injections:				0.020	Pearson chi-square test
No injections	60	0	42		
One injection	110	42	68		
Two injections	39	16	23		
Three injections	10	1	9		
Four or more injections	6	0	6		
Yes (not at Royal Derby Hospital)	21	19	2		
At least one injection (any level of care)	178	78	108	0.099	Pearson chi-square test
Needling:				0.053	Pearson chi-square test
Yes	39	21	18		
No	209	78	131		
Physiotherapy:				0.001	Pearson chi-square test
Yes	134	67	67		
No	107	31	76		
Other related surgeries:				0.811	Pearson chi-square test
Yes	21	9	12		
No	229	92	137		

106 patients had surgery in our study.

### Adjuvant procedures in surgically managed patients

In addition to arthroscopic excision of the calcific deposit, 103 adjuvant procedures were performed. These included: rotator cuff repair (6 patients), subacromial decompression (51 patients), ACJ excision (8 patients) and capsular release (8 patients). These surgical interventions were performed at the same time as the initial arthroscopic debridement. Of the surgically managed patients, 30 went on to have postoperative steroid injections.

### Complications and subsequent operations

Of the 15 surgically managed patients who developed complications, 12 developed adhesive capsulitis with another 3 sustaining rotator cuff tears. Two of the patients who developed adhesive capsulitis went on to have a manipulation under anaesthetic, and one of the rotator cuff tears subsequently required repair. This rotator cuff tear was not present at the time of the initial surgery; however, it became sufficiently symptomatic that it required repair.

Four patients were still symptomatic after their arthroscopic debridement and underwent revision surgery.

Three patients required subsequent procedures, these included one ACJ excision, one subacromial decompression and one patient who underwent both ACJ excision and subacromial decompression. Analysis of the notes of these patients demonstrates that these surgeries were not indicated at the time of the initial arthroscopic debridement of the calcific deposit.

### Outcome measures

Table 4 outlines the OSS and range of movement for operatively managed patients before and after surgery.

**Table 4:** Functional outcome scores in operatively managed patients

	Preoperative mean (SD)	Postoperative mean (SD)	*p*-value (single sample *t*-test)
OSS	22 (8)	35 (11)	<0.001
FF	115 (52)	121 (39)	<0.001
ER	61 (29)	49 (24)	<0.001

ER = external rotation; FF = forward flexion; OSS = Oxford shoulder score; SD = standard deviation

## Discussion

The aim of this study was to determine predictive factors for the failure of nonoperative management of calcific tendonitis. As a secondary outcome, we aimed to verify the previous literature, which has demonstrated that increased calcific deposit size (>10mm) is more likely to require operative intervention.^13,14^

Female patients (*p*=0.005) and those with a calcific deposit larger than 10mm (regardless of sex) (*p*=0.003) were more likely to fail nonoperative management and require operative intervention. Patients receiving treatment in primary care (from a general practitioner and/or a physiotherapist) were more likely to require surgical intervention (*p*=0.001). This was also the case for patients who had symptoms of calcific tendonitis for more than eight months (*p*=0.001) (Table 1). Those who transiently responded to steroid injections were also more likely to require surgical intervention (*p*=0.02).

Female sex was also associated with the failure of nonoperative management. This is consistent with the findings of de Witte *et al*.^18^

Despite sedentary jobs being a previously documented risk factor for the development of calcific tendonitis,^4^ the cohort of patients in this study did not demonstrate any association of occupation or smoking status with the failure of nonoperative treatment.

Although previous studies have shown that diabetes, the location of the calcific deposit, hypercholesterolaemia, hyperlipidaemia, vitamin D level, high BMI, smoking, and occupation type (manual or sedentary) may all dispose to the development, poor healing and severity of the condition,^8,19–22^ our results did not find any correlation between these factors and the failure of nonoperative management

### Calcific deposit

A number of studies in the literature have previously demonstrated that the size of the calcific deposit has no relevance to the severity of the symptoms.^23–25^ Drummond *et al* identified that patients with a calcific lesion over 10mm in size were more likely to fail nonoperative management and require surgery, regardless of its location or patient demographics.^14^ In the large prospective cohort study by Ogon *et al*,^13^ bilateral disease, localization of the calcification to the anterior portion of the acromion, medial extension and increased volume of the calcific deposit were all shown to be poor prognostic factors for nonoperative management. Furthermore, Ogon *et al* demonstrated that Gärtner type III deposits and lack of sonographic sound extinction of the calcification were demonstrated to be positive prognostic factors,^13^ although they did not further assess the results of surgical intervention for these patients or provide any functional scores following management.

Our results confirmed that the size of the calcium deposit is a predictive factor for the failure of nonoperative management. This therefore agrees with the research performed by Drummond *et al*,^14^ and achieves our secondary outcome.

### Predictive symptoms for failure of nonoperative management

In addition to these findings, we identified that patients with a longer chronicity of symptoms and those presenting who had received previous treatments (including physiotherapy) were more likely to require surgery. It is unclear to us why having previous treatments for calcific tendonitis is a prelude to the need for surgery. It may by that it is associated with the chronicity of symptoms. With lengthening NHS waiting times, having previous failed treatment before being reviewed in our clinic could contribute to this association. Additionally, if a patient’s symptoms are severe enough that they have already had failed management previously before being referred to our clinic, it is only natural that surgical intervention is the next step.

Patients who were managed conservatively were found to have had a greater number of steroid injections. It is unclear whether their symptoms were managed adequately by steroid injections or whether, that with increasing waiting times for referral to secondary services, steroid injections were used as a temporising management. Patients who underwent purely nonoperative management had more subacromial injections during their conservative treatment than those who went on to require surgery. Those who received treatment in primary care (from their general practitioner and/or a physiotherapist) were more likely to require surgical intervention.

Arthroscopic debridement is the gold standard procedure for the treatment of calcific tendonitis^16,17^; however, this remains contentious. Its routine use has been criticised due to high rates of postoperative adhesive capsulitis, occurring in approximately 12% of patients.^26^ There is also debate regarding how much of the calcium deposit should be removed,^27,28^ whether rotator cuff repair is required and whether decompression alone is effective as a surgical option.^8,29,30^ Postoperative outcomes have been correlated strongly with the presence of postoperative tendinous calcium deposits regardless of deposit size, shape or location.^30^

Patients undergoing surgical intervention underwent shoulder arthroscopy with excision of the calcific deposit. Only 6 of 106 (5.6%) underwent rotator cuff repair. This is in direct contrast to the findings of Drummond *et al*,^14^ who performed rotator cuff repairs in the majority of their patients (77 of 93), consequently stating that surgical repair may be required regardless of the presence of preoperative cuff tears.

### Complications

Previous studies have shown a low risk of surgical complications postoperatively, which can generally be managed by nonoperative methods.^31^ Out of 106 patients, 15 (14.1%) managed surgically developed complications. This included 12 patients who developed postoperative adhesive capsulitis. Two of these 12 had diabetes mellitus, considered to be a risk factor for the development of adhesive capsulitis.^32^ We did not identify an association between postoperative patients with diabetes developing postoperative adhesive capsulitis.

### Outcome measures

There was no difference between the final (posttreatment) OSS of the patients managed nonoperatively, and those who had undergone surgical intervention for surgical management for nonsurgical management. We would suggest that in those who initially fail nonoperative management, operative intervention remains a good treatment option with good functional outcomes.

Although there was no significant difference at the end of treatment for ER (*p*=0.056), FF was found to be less in the surgical group (*p*=0.031). This could be due to 11.3% of the surgical patients developing adhesive capsulitis postoperatively – a common complication for this type of surgery.^26^ Additionally, as 42.5% of the patients had less than 6 months follow-up, and pain has been shown to continue for an average of 5.7 months after arthroscopic removal,^33^ it may be that, with the short-term follow-up, this study did not adequately capture these data.

### Limitations

Limitations of this study include the short follow-up, final OSS in the nonoperative group and the retrospective time period over which our data were collected. Given the retrospective nature of the study, we were unable to adequately assess quality of life scores and return to work. An additional limitation is the final OSS scores taken for patients. Patients who had operative intervention tended to return to clinic frequently for follow-up consultations in which a repeat OSS could be taken. In the nonoperative group, they were discharged when it was decided that conservative measures had improved their symptoms sufficiently. If we were to do this study again, we would aim to take repeat a OSS a set period of time after finish of treatment.

We are also aware that the data we used were taken between 2014 and 2018. These data were used as it meant we had sufficient follow-up data in these patients. If we were to repeat this study, however, we could use more recent data.

## Conclusions

**Figure 2:**
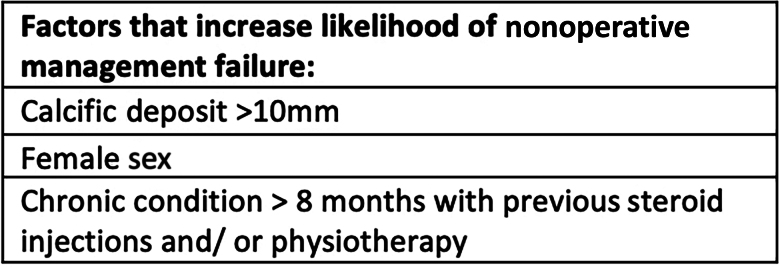
Figure displaying the characteristics and factors that predict the need for operative intervention in calcific tendonitis

This is the first study that reports both the predictive clinical and radiological factors for the failure of nonoperative management in calcific tendonitis. This retrospective review of a large cohort of patients presenting with calcific tendonitis identified that nonoperative management is likely to fail in those with a calcific deposit greater than 10mm on radiographic imaging (confirming previous research), patients of female sex and those who present with symptoms of more than eight months’ duration. Additionally, patients were more likely to require surgery if they had previously had a transient response to steroid injections, and or physiotherapy. Based upon these findings, we were able to create a simple tool to assist clinicians in managing patients presenting with calcific tendonitis, as shown in Figure 2.
